# Garment Recognition and Reconstruction Using Object Simultaneous Localization and Mapping

**DOI:** 10.3390/s24237622

**Published:** 2024-11-28

**Authors:** Yilin Zhang, Koichi Hashimoto

**Affiliations:** Department of System Information Sciences, Graduate School of Information Sciences, Tohoku University, Aoba-ku, Sendai 980-8579, Japan; koichi.hashimoto.a8@tohoku.ac.jp

**Keywords:** instance segmentation, object SLAM, garment reconstruction, robotics, computer vision, clothing manufacturing

## Abstract

The integration of robotics in the garment industry remains relatively limited, primarily due to the challenges in the highly deformable nature of garments. The objective of this study is thus to explore a vision-based garment recognition and environment reconstruction model to facilitate the application of robots in garment processing. Object SLAM (Simultaneous Localization and Mapping) was employed as the core methodology for real-time mapping and tracking. To enable garment detection and reconstruction, two datasets were created: a 2D garment image dataset for instance segmentation model training and a synthetic 3D mesh garment dataset to enhance the DeepSDF (Signed Distance Function) model for generative garment reconstruction. In addition to garment detection, the SLAM system was extended to identify and reconstruct environmental planes, using the CAPE (Cylinder and Plane Extraction) model. The implementation was tested using an Intel Realsense^®^ camera, demonstrating the feasibility of simultaneous garment and plane detection and reconstruction. This study shows improved performance in garment recognition with the 2D instance segmentation models and an enhanced understanding of garment shapes and structures with the DeepSDF model. The integration of CAPE plane detection with SLAM allows for more robust environment reconstruction that is capable of handling multiple objects. The implementation and evaluation of the system highlight its potential for enhancing automation and efficiency in the garment processing industry.

## 1. Introduction

The garment processing industry has been slow to adopt general robotics, in contrast to other sectors that have embraced this technological shift. This hesitation arises from the challenges posed by the highly deformable nature of garments that result in unpredictable shapes, which complicates robotic perception, generalization, and manipulation. Existing research in robotic handling of deformable objects [1,2,3] has largely been limited to controlled environments, addressing specific objects or operations. Consequently, there is a pressing need for a versatile garment-handling robot that can handle diverse garment types and adapt to varied working conditions. One possible way to achieve this is through the development of a specialized vision-based model for detecting and reconstructing garments in 3D.

Vision-based methods for garment handling robots have demonstrated potentials but face significant limitations. Traditional 2D approaches [4,5,6] focus on detecting garment features such as wrinkles or edges to infer grasping points. However, these methods often suffer from inefficiencies, such as repetitive grasping attempts to fully open garments [4], or are constrained to overhead views with limited environmental adaptability [3]. Refs. [5,6] utilize a learning-based approach directly predicting two grasping points for each defined action from an RGBD (RGB-Depth) image. However, they are also constrained to overhead view and limited to single t-shirt handling. Other recent works have made advances in 3D garment perception. Ref. [7] aligns point clouds with garment images and derives grasping points through simulation, but its application is limited to slightly crumpled single garments. Ref. [8] reconstructs garment meshes from depth images for use in robotic action planning; however, it is restricted to inconsistent, static observations. Ref. [9] employs deep learning to establish mesh correspondences and infer 3D geometry from images, yet it is constrained to single-layer fabrics and relies solely on static observations. While these techniques improve geometric understanding, their constraints make them unsuitable for dynamic industrial environments. These limitations highlight a broader issue in garment-handling robotics: the reliance on constrained environments with fixed viewpoints and restricted fields of view; lack of perception of the operating environment comprehensively; and the inability to process consistent frames in dynamic settings in 3D.

Beyond garment handling, 3D sensing and reconstruction techniques [10,11,12,13,14,15] have seen widespread application in fields like crack damage recognition, agriculture, mining, autonomous driving, and virtual/augmented reality. However, their adoption in the garment industry remains rare. Additionally, advancements in Simultaneous Localization and Mapping (SLAM) with object or plane integration [16,17] provide promising solutions for mapping and understanding complex environments. Ref. [16] incorporates cuboid objects and planes into SLAM’s joint optimization and reconstructs a dense map. Ref. [17] builds a lightweight and object-oriented map with general models, which can be integrated into robotic grasping tasks with its object parameterizations, but the objects are also represented by cuboids and spheres. These methods’ limitations to rigid cuboid or sphere representations hinder their ability to accurately model the deformable and irregular surfaces of garments and to simulate garment interactions effectively. Furthermore, dense maps generated by these SLAM techniques often result in incomplete object reconstructions, and the use of prior object models can lead to discrepancies between the model and the actual object, making them unsuitable for garments with highly variable shapes.

This work seeks to address these limitations by implementing a vision-based model for garment-handling robots. The proposed approach incorporates garment detection and 3D reconstruction within an object-oriented SLAM framework. Specifically, it leverages 2D instance segmentation for garment detection, integrates plane detection for environment understanding, and utilizes a learning-based shape prediction algorithm to reconstruct the detected garments from the SLAM mapping points. This model aims to overcome the constraints of 2D-only perception, static observation, and rigid object representations, enabling real-time garment and environment reconstruction suitable for dynamic industrial scenarios. To support models’ developments, a 2D garment image dataset and a 3D garment mesh dataset are collected and manually annotated, facilitating robust training for segmentation and reconstruction tasks. Experimental results demonstrate improved model performance, showcasing the feasibility of simultaneous garment and plane detection and reconstruction using the integrated SLAM framework. In this study, we focus primarily on T-shirts in industrial environments as a foundational step, setting the groundwork for extending the framework to more complex garment types and dynamic scenarios in future research.

To summarize, the following contributions are presented in this paper:

- An object-oriented SLAM that incorporates plane detection is proposed, which includes planes in the joint optimization process and reconstructs them to facilitate understanding of the environment.
- The proposed SLAM-based method is tailored specifically to address the challenges of garment recognition and reconstruction, accommodating the highly deformable and irregular nature of garments.
- Six common garment states observed in industrial processes are identified and defined, providing a semantic basis for understanding and processing garments in various conditions. And segmentation models and mesh reconstruction models are trained using datasets collected and annotated based on the defined garment states.

The subsequent sections of this paper are organized as follows. Section 2 provides an overview of related work. Section 3 explains the methodologies of the integrated SLAM, while Section 4 presents the experimental procedures and outcomes. Discussion of the results and outlines for future improvements is elaborated in Section 5. Finally, Section 6 offers a conclusive summary of this work.

## 2. Related Works

SLAM (Simultaneous Localization and Mapping), particularly visual SLAM, is a common method in robotics enabling devices to comprehend their position in unknown environments. It leverages visual data to generate cost-effective information. ORB-SLAM2 [18], an advancement over ORB-SLAM (Oriented FAST and Rotated BRIEF SLAM) [19], efficiently uses camera data for real-time mapping, representing environments via keyframe graphs. The limitations of point-feature-focused SLAM in low-texture environments spurred the development of feature-diverse methods like PlanarSLAM [20]. CAPE (Cylinder and Plane Extraction) [21] also exhibits the potential to aid SLAM by feature extractions from depth camera data. To improve environment understanding by incorporating semantic and object-level information, segmentations are added as a new feature in SLAM. However, the sparse point cloud typically obtained through SLAM does not provide sufficient support for 3D instance segmentation while dense point map often struggles with loop closure and speed. Thus, using 2D segmentation, subsequently projecting the result to 3D space, and utilizing plane features could be advantageous. DSP-SLAM (SLAM with Deep Shape Priors) [22] is one such method, utilizing ORB-SLAM2 for tracking, mapping, Mask R-CNN [23] for instance segmentation, and DeepSDF [24] for object reconstruction. DeepSDF is an example of reconstructing rigid objects using mesh, which represents a 3D shape through a continuous Signed Distance Function (SDF) learned by a deep neural network. These studies provide valuable insights for the research field and for this work.

## 3. Materials and Methods

### 3.1. System Overview

**Figure 1:**
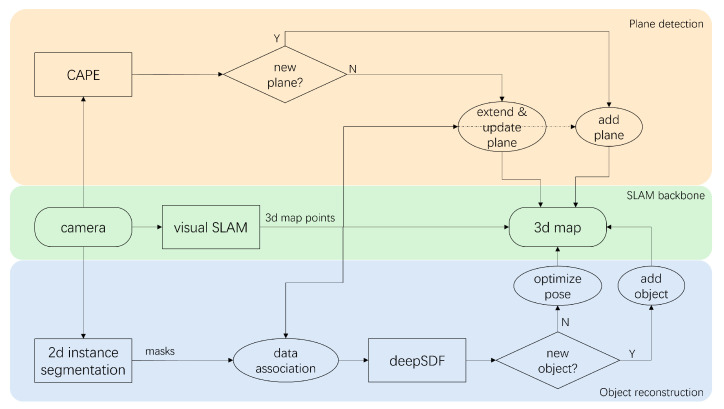
Work flow of the integrated SLAM. The SLAM backbone detects feature points and reconstructs a 3D map of sparse points. The object reconstruction thread detects garments and desks and reconstructs them by DeepSDF [24] in the map. The plane detection thread detects and reconstructs planes using CAPE [21] and matches them to the map.

An overview of the integrated SLAM system is depicted in Figure 1. The system can be divided into three threads, with ORB-SLAM2 serving as the foundational SLAM backbone. The object thread aiming to detect and reconstruct garments follows the idea of DSP-SLAM, which performs 2D instance segmentation on each keyframe’s RGB image and then reconstructs segmented objects by DeepSDF from the masks inferred and the 3D map points derived from SLAM. Each object will either be associated with existing objects in the map to update the pose or be initialized and added as a new object. Simultaneously, in the plane thread that aims to detect and reconstruct important environmental planes such as walls and table surfaces and improve SLAM’s performance in low-textured environments, depth images from each frame are fed into CAPE, and detected planes are either associated with existing planes in the map to extend them or added as new entities. In the following subsections, implementation details will be introduced.

### 3.2. Garment Detection

To develop an instance segmentation model proficient in identifying garments within a particular environment and understanding the condition they are in, a dataset of various garments has been created and manually annotated. In terms of garment type, the focus at this stage is primarily on t-shirts, driven by the fact that one of the main example tasks of our study is the process of t-shirt printing.

**Figure 2:**
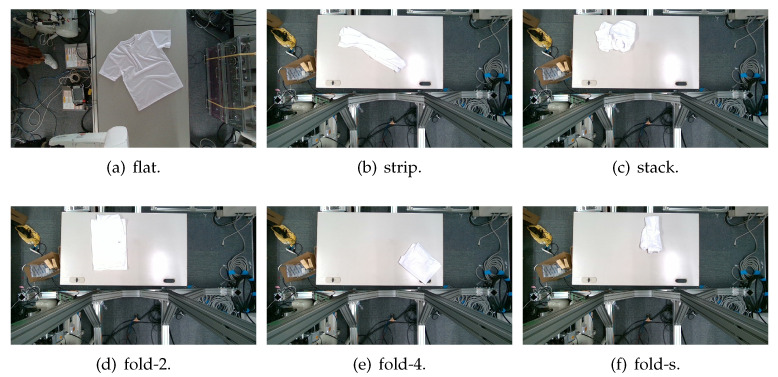
Examples of the 6 garment states.

The garments in our study are categorized into six different states based on practical reasoning, as illustrated in Figure 2, taking into account the most frequent conditions a t-shirt might assume throughout the industrial processing. This classification stems from common real-life actions and observations made at a garment factory, as follows:

- **Flat**: This state denotes a t-shirt laid out largely flat on a surface, maintaining its recognizable shape. It may exhibit minor folds, particularly at the corners of the cuff or hem and sleeves.
- **Strip**: In this state, the t-shirt is folded vertically but spread horizontally, assuming a strip-like shape. This can occur either by casually picking up the garment around the collar or both shoulders and then placing and dragging it on the table, or by carefully folding it from the flat state.
- **Stack**: This state occurs when the t-shirt is picked up randomly at one or two points and then casually dropped on the table.
- 2-fold (or **fold-2** as referred to later): Here, the t-shirt is folded once horizontally from the flat state, with the positions of the sleeves being random.
- 4-fold (or **fold-4** as referred to later): In this state, the t-shirt is folded into a square-like shape. This can be achieved either by folding one more time vertically from the 2-fold state, or by folding in the style of a “dress shirt fold” or “military fold”.
- Strip-fold (or **fold-s** as referred to later): Here, the t-shirt is folded once more horizontally from the strip state. This mimics the method typically employed when one wishes to fold a t-shirt swiftly and casually.

The orientation of the t-shirt, whether it is front-up or back-up, is not predetermined but random. All the images in this dataset have been manually annotated by outlining each t-shirt segment and assigning a state class using LabelMe [25].

Our study encompasses four different versions of the dataset and each includes a separate set of test sets that emphasize the unique characteristics of the respective datasets:

1. **V1** comprises 200 RGB images of a single t-shirt exhibiting painted sections, inclusive of both long and short-sleeved variants. The camera is strategically positioned above the table, capturing a bird’s-eye view of the garment and the table beneath it.
2. **V2** adds colored and strip-patterned t-shirts. The test set featuring these new images is denoted as **v2c** in subsequent references.
3. **V3** features multiple t-shirts (ranging from 2 to 6) within the same frame. The general test set featuring v3’s new data is identified as **v3m**. These images can be further divided into various sets based on the interaction among the t-shirts: (1) **v3ma**: several t-shirts of identical or differing colors placed separate from each other. (2) **v3mcd**: numerous t-shirts of varying colors placed adjacent to each other. (3) **v3mcs**: several t-shirts of the same color positioned right next to each other, with some arranged intentionally to create confusion. V3mcs type is only used for testing purposes, and is not included in the training set.
4. **V4**’s test set is referred to as **v4sd**. Two elements undergo change or addition: (1) camera view: the camera is no longer static over the table’s top. Instead, the images are derived from videos where the camera’s movements mimic a mobile robot, focusing on the operating table with the garments placed on it. (2) A new object class: the operating table is introduced as the 7th class in the detection.

**Figure 3:**
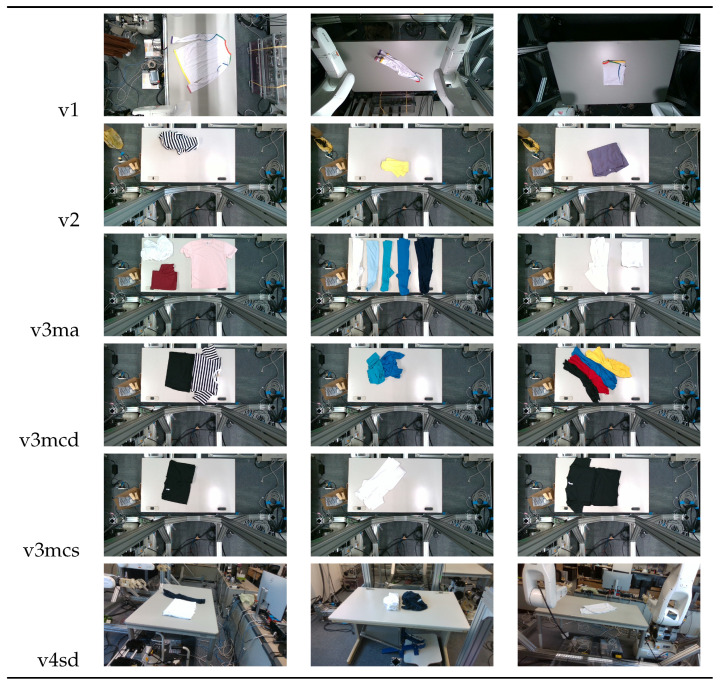
Example images from different version’s dataset.

A summary of the four versions of the dataset and their corresponding test sets is provided in Table 1. Examples of each test set’s data are listed in Figure 3.

Current 2D instance segmentation models can be broadly divided into two categories—two-stage approaches and one-stage approaches. While the two-stage approaches often deliver superior accuracy, the one-stage approaches distinguish themselves with their rapid detection speeds. Given the necessity for real-time operation and a balanced performance between precision and speed, this study employs four different models for training and testing purposes. Following an evaluation of the test results, a single model is selected. The four candidate models include MASK-RCNN [23] (employed as a baseline for comparison), YOLACT [26] (You Only Look At CoefficienTs), SOLOv2 [27] (Segmenting Objects by Locations), and SOLOv2-light (serving as a speed-accuracy trade-off comparison for SOLOv2). The models were selected based on the runtime statistics presented in their original research papers.

The selected models utilize RGB images as inputs and generate outputs in the form of bounding boxes *B*, represented by the coordinates of diagonal points $\left(x_{1},y_{1},x_{2},y_{2}\right)$, the label of object classes ranging from 0 to 5 or 0 to 6 in the case of dataset v4, and masks *M* represented by the coordinates of polygon vertices. For single-stage detectors, bounding boxes are constructed based on the marginal vertices of the masks. The labels and masks are subsequently employed to identify the 3D points associated with the objects, facilitating their reconstruction on the map, a process to be detailed in Section 3.5. The model parameters are refined and fine-tuned during the training phase.

### 3.3. Garment Reconstruction

Following the DSP-SLAM [22] methodology, DeepSDF [24] is employed as the object reconstruction method. DeepSDF is not only adept at shape reconstruction and completion tasks but also exhibits potent shape interpolation capabilities. This enables it to offer a more flexible reconstruction that adheres to the object’s form with minimal prior knowledge (training data), as opposed to constructing a rigid mesh solely based on prior knowledge and the identified object class.

In the context of this study, DeepSDF generates the SDF value s=G(x,z) by taking as inputs a shape code *z* and a 3D query location *x*.

In order to train a DeepSDF model capable of reconstructing garment meshes in six distinctive states and aligning with the instance segmentation results, a custom garment mesh model dataset is manually crafted. For this process, we employ Blender 3.4 [28], a free and open-source 3D computer graphics software toolkit widely used for various purposes. Blender’s parameters are calibrated through a series of experiments to fine-tune aspects such as the appearance and the motion performance, to obtain more realistic characteristics including the garment’s wrinkles.

The resulting dataset comprises 44 garment mesh models in total (4 flat, 12 strip, 9 stack, 4 fold-2, 10 fold-4, 5 fold-s), alongside 4 desk models drawn from ShapeNet v2 [29]. Sample meshes generated from this dataset are depicted in Table 2.

### 3.4. Plane Detection and Reconstruction

In this work, CAPE [21] is utilized to extract plane features. CAPE’s plane extraction starts from a plane segmentation step conducted by region growing from randomly sampled points on the depth image. Plane models are subsequently fit to the plane segments. And finally refinement steps are performed to optimize the detected planes. The output planes $\pi_{c}$ of CAPE are represented by a Hesse normal form $\pi_{c}=\left(n,d\right)$, where $n=(n_{x},n_{y},n_{z})$ denotes the unit normal vector of the plane, and *d* denotes the distance from the origin of the coordinate system to the plane, with both pieces of information described in the camera coordinate system.

To determine the plane’s position in the world coordinate system, the transformation matrix $T_{cw}$ which conveys the transformation from world coordinate system to the current camera coordinate system is required, and it can be obtained during the SLAM process. The plane can thus be represented by

(1) $$\pi_{c}=T_{cw}^{-T}\pi_{w}\;.$$

However, Hesse normal form plane π cannot be directly used in the SLAM system—specifically, during the bundle adjustment process—for estimating camera pose due to over-parameterization. In this work, coordinate system transformation is considered to solve this issue. In the Hesse normal form of a plane π=(n,d), *d* can be viewed as a distance or length, while $n=(n_{x},n_{y},n_{z})$ can be viewed as a unit normal vector that represents the orientation of the plane and this is where the redundancy originates. Spherical coordinate system is leveraged, using two angles θ and φ to represent a unit vector in the 3D space, and with θ as the azimuth angle and φ as the elevation angle, the Hesse normal form plane π can be reformed into the following:

(2) $$\pi_{s}=\left(\varphi ,\theta ,d\right),where\left\{\begin{array}{c} \varphi =\arctan\frac{n_{y}}{n_{x}}\\ \theta =\arcsin n_{z} \end{array}\right..$$

Notice that θ and φ have a limited range of (−π,π) to circumvent singularity problems.

### 3.5. SLAM Integration

#### 3.5.1. Object Reconstruction and Association

After the instance segmentation, a bounding box *B*, a mask *M*, and a class label *C* will be assigned to each candidate instance *I*. And from the SLAM backbone, a set of sparse point observations *D* is obtained. The task is then to estimate the dense shape *z* and its 7-dof pose $T_{co}$ from these. The initial pose $T_{co,0}$ is obtained by applying PCA to the sparse point cloud of the object and initial shape code z=0. Then *z* and $T_{co}$ are refined iteratively as a joint optimization problem.

Two loss terms in terms of energy term [22] are initially used in this refinement procedure. Surface Consistency Loss $L_{surf}$ measures 3D points’ alignment with the surface of the reconstructed object, and differentiable SDF Render Loss $L_{rend}$ calculates a difference between the observed depth and expected depth to align the size.

In order to reconstruct multiple objects, especially in the case where two objects are overlapping in the camera view, the above-mentioned refinement is conducted for every detected instance. In addition, in the process of calculating the occupancy probability of whether a point is inside, outside, or on an object, every point will be recalculated for the second object even if it’s already calculated to be inside one object in case of occlusion.

However, these would lead to another issue: penetration or collision between reconstructed objects. To mitigate this, we introduce a Repulsive Loss $L_{repu}$ [30] into the overall loss function. Penetrations are discerned by the number of times a ray from a point on one object intersects the surface of another object. And Repulsive Loss calculates the distances of all the penetrated sampled points to another object. Unfortunately, this loss is still not ideal because of the randomness of objects’ poses and the limited number of rays and sampled points; the projected rays will not always detect existing penetrations. This is to be improved in the future.

The final loss *L* is a weighted sum of the Surface Consistency Loss $L_{surf}$, the SDF Render Loss $L_{rend}$, Repulsive Loss $L_{repu}$, and a regularization term of shape *z* with their respective weights ($\lambda_{s}$, $\lambda_{r}$, $\lambda_{R}$, and $\lambda_{c}$):

(3) $$L=\lambda_{s}L_{surf}+\lambda_{r}L_{rend}+\lambda_{R}L_{repu}+\lambda_{c}{∥z∥}^{2}\;.$$

**Figure 4:**
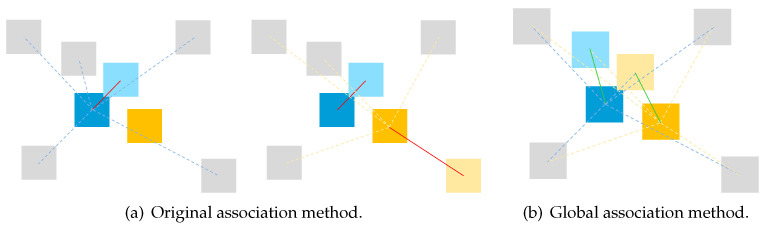
Comparison of a potential false association case with the correct result by the global association method. In situations where two objects, A and B, are in close proximity, B’s true match might be misidentified as A’s closest candidate by the original method, leading to its removal from B’s candidate list. A global association method, however, could correctly associate the objects.

Once detections are reconstructed, they are to be associated with objects on the map. The number of matched feature points $N_{d}$ between the detection and the object is the criteria. If $N_{d}$ is bigger than a threshold $N_{th}$, then the detection will be considered an association candidate. In [22], only the nearest is maintained if one object might also be assigned multiple detection candidates. However, this could lead to incorrect associations in cases where multiple objects are in close proximity and cameras are moving quickly, and when one detection might be the association candidate for multiple objects, as depicted in Figure 4. To resolve this, a global minimal association distance of all the association candidates is calculated instead.

Detections that are not the association candidates for any existing object will be considered new object candidates. To mitigate the impact of erroneous detections and outliers, a candidate for a new object will only become a true new object and be added into the map when it is detected in $N_{kf}=5$ consecutive keyframes.

#### 3.5.2. Plane Matching

The plane feature matching can be conducted by comparing the angle and distance between the two planes, which can be obtained by calculating from the distance *d*, and the normal vector *n* or the angles (θ,φ) under the spherical coordinate system. Firstly, the angle between the two planes $\Phi_{p}$ is calculated. And only when $\Phi_{p}<\frac{\pi }{6}$, the distance $d_{p}$ between the two planes will be calculated. The distance $d_{p}$ is calculated by

(4) $$d_{p}=\frac{1}{m}\sum_{i=0}^{m-1}d\left(p_{i},P_{c}\right)\;,$$

where $p_{i}$ is the *m* randomly sampled points from the map plane, and $P_{c}$ is the detected plane in the current frame [31]. If $d_{p}<0.1\left(m\right)$, the detected plane will be considered a match for the map plane.

For the matched planes, new point clouds are built and extended into the matching map planes. The detected planes that are not matched to any existing map plane will be considered new planes and added into the map. The Voxel grid in PCL library is used to filter the plane point cloud, which helps reduce the number of points and computation cost. The leaf size of voxel grid is set to be 0.05.

Similar to object association, in order to reduce the number of bad planes and noises, every newly detected plane has to be detected more than n=0.2∗FPS times to be added to the map.

#### 3.5.3. Joint Optimization

Keyframe acceptance rules are modified because of the addition of plane features in each frame. The frame that detects a new plane will be considered a keyframe and will not be removed as bad keyframe in the local mapping thread. In addition, plane features are also considered in the tracking initialization. Other than detecting more than 500 point features, detecting no less than three planes and at least 50 points is also considered feasible for initialization. The numbers are given by experiments.

As the baseline method, ORB-SLAM2 uses Bundle Adjustment to optimize the camera pose and the map of points. In this work, the final map consists of a set of camera poses $C=T_{wc_{i}}$, map points $D=p_{j}$, object poses $O=T_{wo_{k}}$, and map planes $P=\pi_{w_{l}}$. And they can be optimized by a joint BA as a least squares optimization problem following the same idea, as follows:

(5) $$C*,D*,O*,P*=\arg\min\sum_{i,j}{∥e_{cd}\left(T_{wc_{i}},p_{j}\right)∥}_{\sum_{i,j}}+\sum_{i,k}{∥e_{co}\left(T_{wc_{i}},T_{wo_{k}}\right)∥}_{\sum_{i,k}}+\sum_{i,l}{∥e_{cp}\left(T_{wc_{i}},\pi_{wl}\right)∥}_{\sum_{i,l}}\;,$$

where ∑ represents the covariance matrix, and $e_{cd}$, $e_{co}$, and $e_{cp}$ represent the error equation between the camera pose and the map points, object pose, and map planes, respectively. Camera-point error $e_{cd}$ is the same as the reprojection error in ORB-SLAM2. Camera-object error $e_{co}$ follows that of DSP-SLAM, while camera-plane error $e_{cp}$ follows the same pattern as camera-point error, as follows:

(6) $$e_{cp}=T_{cw}^{-T}\pi_{wl}-\pi_{l}\;,$$

where $\pi_{wl}$ is the matched map plane in the world coordinated system of plane $\pi_{l}$ in the camera coordinate system.

**Figure 5:**
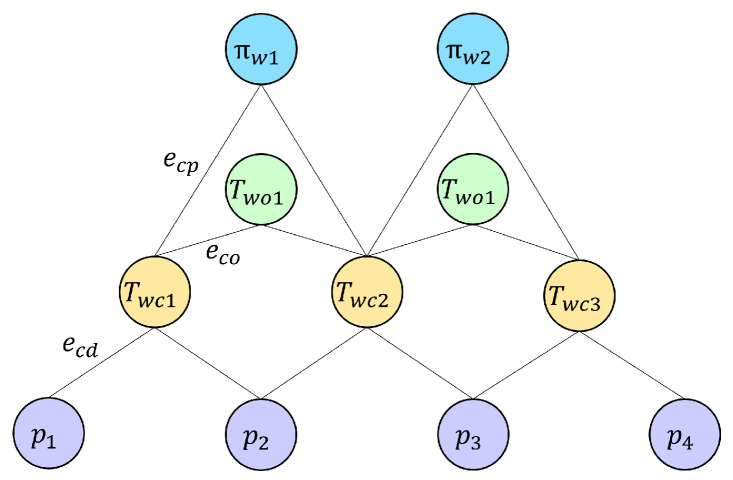
Illustration of graph optimization. Orange vertices represent camera poses, purple vertices represent feature points, green vertices represent object poses, and blue vertices represent planes. The edges connecting these vertices represent the error terms to be optimized.

Similar to ORB-SLAM2, this BA problem is constructed as a graph optimization, and both the objects and planes are treated as vertices like the feature points, as dipicted in Figure 5. The error terms, which can be seen as new pose observations, serve as edges in the graph connecting camera pose vertices to feature point vertices, object pose vertices, or plane vertices.

## 4. Results

### 4.1. 2D Garment Recognition

This subsection delves into the experiments conducted using four instance segmentation models (MaskRCNN, YOLACT, SOLOv2, SOLOv2-light) trained on various versions of our collected garment dataset and tested on different test sets emphasizing various features. The underlying expectation was that the custom dataset would enable the models to proficiently recognize garments (t-shirts) and the environment (desk) with diverse attributes. Subsequently, based on the evaluation of segmentation accuracy and inference speed, one model was selected for integration with the SLAM framework. All training and testing processes were performed on an Intel CORE i9 CPU paired with an NVIDIA RTX 3090 laptop GPU (Intel: Santa Clara, CA, USA; NVIDIA: Santa Clara, CA, USA).

**Figure 6:**
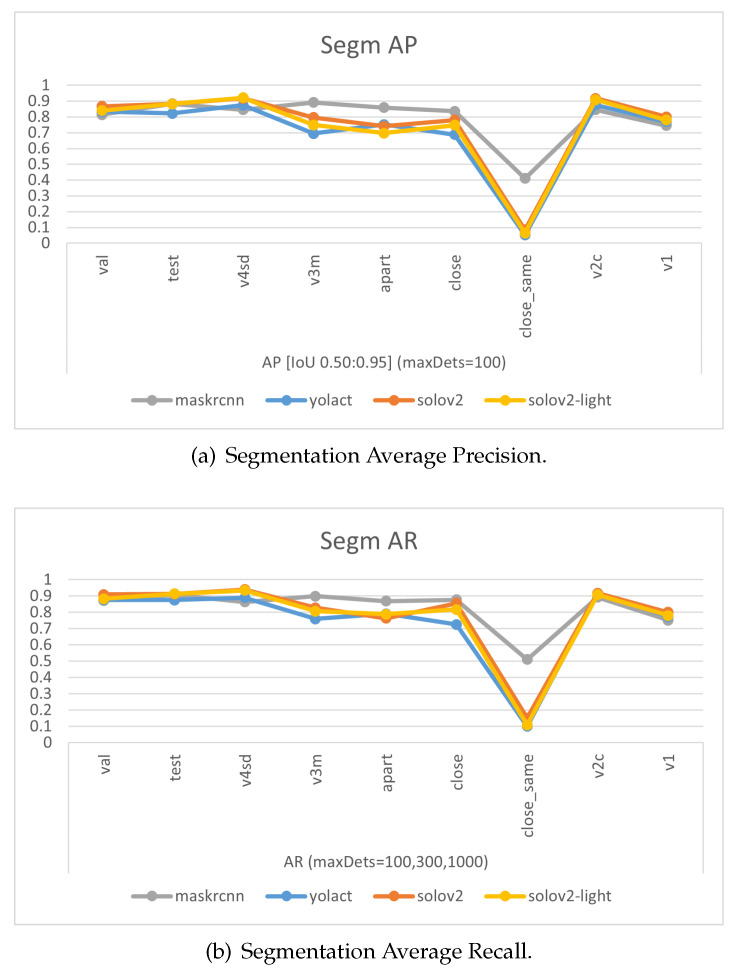
Charts of Average Precision (AP) and Average Recall (AR) evaluated on different test sets for the four models.

The experimental results of models trained on the final dataset v4 emphasize that overall, as shown in Figure 6 and Table 3 and Table 4, SOLOv2, and SOLOv2-light outshine YOLACT and Mask R-CNN in terms of the precision of their masks. Notably, MASK-RCNN displays superior accuracy over the other three models when it comes to scenarios with multiple garments. Especially in situations where the data are unfamiliar—instances of v3mcs that were not included in the training dataset—MASK-RCNN exhibits a slightly stronger generalization ability. However, for single-garment scenarios, or those involving a shift in viewing angle or the addition of the ‘desk’ class, the performance ranks as follows: SOLOv2 >= SOLOv2-light > YOLACT > Mask-RCNN. Interestingly, the overall trend of Recall closely mirrors that of Average Precision (AP). This consistency between recall and precision suggests that the trained models are well-balanced, showing similar tendencies in identifying relevant instances (recall) and correctly labeling instances as relevant (precision). It reflects a situation where the model has a good equilibrium between its ability to find all the relevant instances and its ability to minimize the incorrect classification of irrelevant instances, thus demonstrating both effective and reliable performance.

**Figure 7:**
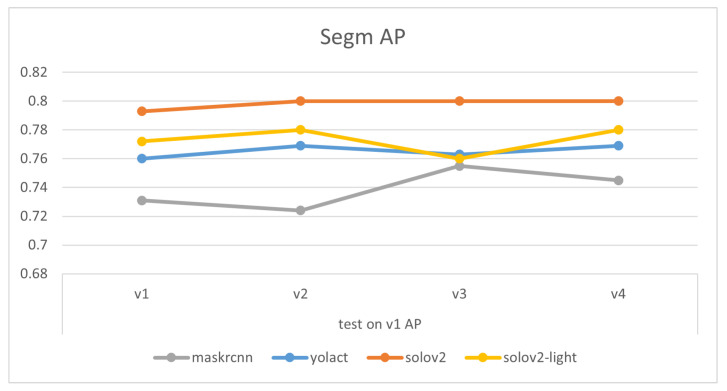
Variations in models’ performances trained on different datasets, tested on v1.

**Figure 8:**
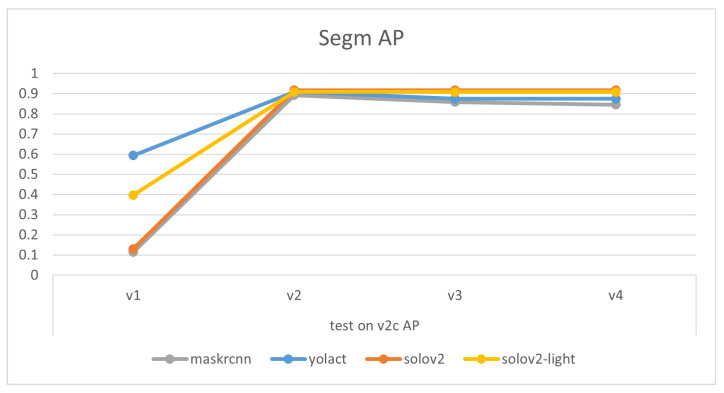
Variations in models’ performances trained on different datasets, tested on v2c.

**Figure 9:**
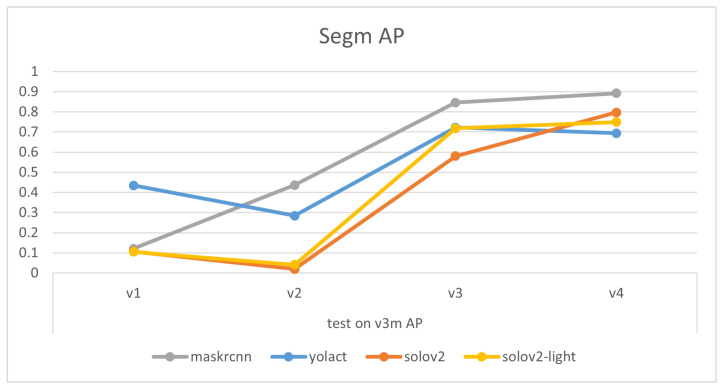
Variations in models’ performances trained on different datasets, tested on v3m.

**Figure 10:**
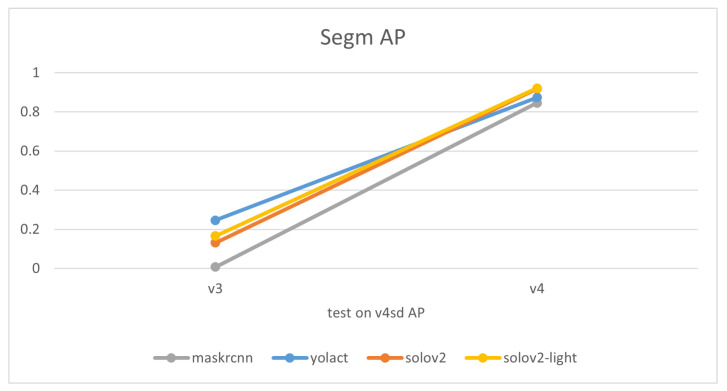
Variations in models’ performances trained on different datasets, tested on v4sd.

Comparisons of models’ mask precision throughout the addition of new training data, as illustrated in Figure 7, Figure 8, Figure 9 and Figure 10, demonstrate that generally, the expansion of the dataset improves models’ performances. Distinctly, the addition of v4’s new data led to a decrease in the accuracy of YOLACT’s recognition of the v3m dataset. This could be because YOLACT was ‘overfitting’ to multiple objects with similar sizes. On another note, YOLACT has better precision on untrained data type, e.g., tested on v4sd when only trained on v3, which implies that it has better generalization capabilities.

Examples of visualized estimated masks are demonstrated in Table 5 and Table 6. It is worth noting that, while SOLOv2 demonstrates satisfactory performance in recognizing and distinguishing multiple objects such as tables and clothing, it faces difficulties in identifying multiple pieces of clothing. Although it maintains the highest mask margin precision for all the test sets, which corresponds to the high AP evaluated, SOLOv2 struggles to tell different garments apart. This may be attributed to its nature as a location-based one-stage method, thereby limiting its capacity to discern multiple similar objects like garments. On the other hand, while YOLACT performs nicely on distinguishing multiple garments, it sometimes struggles with mask boundaries when garments are touching.

The models were also tested with a sequence of RGB images of resolution 1280 × 720, recorded by an Intel Realsense D435i camera. The camera’s movements were designed to imitate the motion needed to scan the environment, with the operating desk and garments roughly in the center of view. This mimics a real-world scenario, creating conditions for our models that reflect those they would encounter in practical applications. Example results are as presented in Table 6. Mask-RCNN faces challenges in recognizing the desk, and the masks of garments lack precision as with the dataset. YOLACT, on the other hand, demonstrates an ability to accurately segment both the garments and the desk, despite occasional difficulties with an edge of the desk. SOLOv2 and SOLOv2-light present the most accurate desk masks, but they still struggle to differentiate between separate garments.

Furthermore, the models are tested directly using an Intel Realsense D435i camera to run in real-time. Input RGB frames are with sizes of 1280 × 720. The example results are as presented in Table 6. The performances are similar to those of the recorded videos. YOLACT demonstrates a superior balance in the precision of mask margins and the differentiation of individual garments. Interestingly, YOLACT, SOLOv2, and SOLOv2-light are capable of detecting the partially visible second desk in the frame, even though such situations were not included in the training set. However, only YOLACT correctly identifies it as a separate desk; SOLOv2 and SOLOv2-light struggle to differentiate between individual objects and incorrectly perceive it as part of the central desk.

In the context of speed, SOLOv2-light leads the pack for recorded videos, while YOLACT emerges as the fastest when tested with camera real-time, as demonstrated in Table 7.

In summary, the performance of the models can be ranked differently depending on the scenario at hand, as follows:

- **Single t-shirt.** For the detection of a single clothing item, the models are ranked based on mask precision as follows: SOLOv2 >= SOLOv2-light > YOLACT > Mask-RCNN. The primary distinction in this scenario is the accuracy of mask edges.
- **Multiple t-shirts.** When it comes to detecting multiple clothing items, the rankings shift to: Mask-RCNN > YOLACT >> SOLOv2-light >= SOLOv2, the main distinction lying in the models’ ability to differentiate between different pieces of clothing. Notably, even though SOLOv2 and SOLOv2-light yield the most accurate masks among the four models, in most cases, they struggle to distinguish different t-shirt pieces. This discrepancy may contribute to their higher rankings when only considering mask precision on the v3m test set, where the ranking is Mask-RCNN > SOLOv2 > SOLOv2-light > YOLACT.
- **Shifting view angle.** In the context of recognizing objects from various angles and identifying desks, the ranking shifts to SOLOv2-light >= SOLOv2 > YOLACT > Mask-RCNN.
- **Reference speed.** Finally, in terms of inference speed, SOLOv2-light and YOLACT are the fastest, trailed by Mask-RCNN and SOLOv2.

Considering all the results, YOLACT stands out as the model that strikes a good balance in all categories and performs commendably across all the tested scenarios. Hence, YOLACT was chosen for further experimentation and SLAM integration.

### 4.2. Garment Mesh Reconstruction

DeepSDF were trained and tested on the self-created garment mesh dataset to enable the model of reconstructing garments under various states. The reconstructions were evaluated by Chamfer distance, which was calculated by determining the average Euclidean distance of each point on the reconstructed mesh to its nearest point on the ground truth mesh, and of each point on the ground truth mesh to its nearest point on the reconstructed mesh. The results of the DeepSDF reconstruction are presented in Table 8 and Table 9. At present, objects with more pronounced three-dimensional features tend to have better reconstruction results. The hierarchy of reconstruction accuracy appears to follow this order: tables show superior results, followed by stacks, and finally, other flatter garment states. It is worth noting that unfolded sleeves present significant ambiguity and currently occupy a disproportionately large portion of our dataset. Future work could aim at better distribution of garment states in the dataset to address this issue.

### 4.3. SLAM Integration

This subsection provides an overview of the experiments conducted on open-source datasets and real-time data, respectively, to test the feasibility of this integrated SLAM approach. To highlight the impact of integrated plane detection and object reconstruction functions on our system, tests were carried out on versions of the SLAM system: one that integrates only the object reconstruction, and another one that integrates only the plane detection. We will compare and contrast these results to provide a view of the different systems’ performances.

Firstly, only the object reconstruction component was introduced into the SLAM system (akin to DSP-SLAM). Utilizing a MaskRCNN model trained on the COCO dataset [32] (Common Objects in Context), tests were conducted and object reconstruction was performed on the Redwood dataset [33]. The camera trajectory error was then evaluated on the KITTI dataset [34]. A Relative Translation Error (RTE) and a Relative Rotation Error (RRE) of 1.41 and 0.22 were achieved, which unfortunately could not outperform ORB-SLAM2’s 1.38 and 0.20.

Subsequently, only the plane detection component was integrated into the SLAM system and comparisons were carried out with ORBSLAM2 using the TUM dataset [35]. On the sequence fr3_cabinet where ORB-SLAM failed to track due to insufficiency of feature points, integrating the plane thread achieved an absolute trajectory error (ATE) of 0.0202.

The SLAM systems were then tested using an Intel Realsense D435i camera as the input sensor capturing RGB and depth images with a size of 680 × 480. The systems ran on a laptop with an Intel i9 CPU and an NVIDIA RTX 3090 Laptop GPU. The results show that this SLAM-based approach could successfully detect and reconstruct the garments and large environmental planes, as presented in Table 10. However, several challenges are evident: the quality of reconstruction and the execution speed. The reconstructed garments do not always accurately represent their real-world counterparts, and penetrations still occasionally occur. This could be attributed to both the reconstruction loss function and the mesh dataset. Regarding the speed, a decrease can be observed with the addition of each detection model, as demonstrated in Table 11. Thus, an improved strategy for computing is necessary.

## 5. Discussion

The results of the instance segmentation analysis demonstrate that the models trained on our collected dataset exhibit strong performance, achieving an Average Precision of 0.882 and an Average Recall of 0.913. These metrics confirm the effectiveness of the dataset in capturing key garment states and shapes. While the current dataset focuses on six garment states, expanding it to include additional garment types and states, particularly those with complex textures or folds, is a priority for future work. Leveraging the garment state information could further enhance robotic operations by enabling grasp point prediction and garment part estimation, potentially reducing task completion time. However, a notable limitation is the model’s difficulty in distinguishing multiple garments of the same color when stacked, which requires both dataset augmentation and model architectural improvements.

The integration of SLAM with garment and plane detection demonstrated promising results. The system achieved an absolute trajectory error of 0.0202 on sequences from the TUM dataset where SLAM without plane detection fails. This highlights the benefit of incorporating plane reconstruction for handling structured industrial environments. However, challenges remain in improving the reconstruction quality. The DeepSDF model achieved an average Chamfer distance of 0.000998, but its performance was limited when reconstructing flatter garments or garments with complex shapes. This may be attributed to biases in the training dataset, where unfolded sleeves dominate, or to the limited number of input points extracted by SLAM. Enhancing the dataset to include more diverse garment geometries and increasing the number of input points are potential solutions.

Additionally, the system’s computational performance requires optimization. Our analysis revealed that GPU utilization remained below 30% during processing, while the CPU frequently operated at full capacity. Future work could explore parallel computing strategies to distribute tasks more efficiently across CPU cores and improve processing speed.

## 6. Conclusions

This study proposed and evaluated an object SLAM-based approach for garment recognition and environment reconstruction, aimed at aiding robotic operations in garment processing tasks. The key findings of this work include the following:

- Instance segmentation models trained on our dataset achieved strong performance, with an Average Precision of 0.882 and an Average Recall of 0.913, demonstrating their effectiveness in recognizing garment states and shapes.
- The DeepSDF-based garment reconstruction achieved an average Chamfer distance of 0.000998, successfully reconstructing garments but showing limitations with flatter or more complex shapes.
- The integrated SLAM system achieved an absolute trajectory error of 0.0202 in sequences with a small number of point features, highlighting its feasibility in industrial environments with plane reconstruction.

Despite these achievements, several limitations were identified. The dataset is currently limited in diversity, and future work should focus on augmenting it to include more types of garments and interactions of the garments, as well as improving the model architecture to handle challenges such as overlapping garments and the same colors. Additionally, the computational performance of the system requires optimization, particularly in CPU task distribution and parallelization.

This work lays a foundation for future research and practical deployment in the garment processing industry. Expanding the system to handle diverse garment types and more complex environments, while improving computational efficiency, will be essential for transitioning this technology from the laboratory to real-world industrial settings. Addressing challenges in technology transfer, such as integrating this system into existing workflows, will further enhance its applicability and impact.

## Figures and Tables

**Table 1:** Summary of the four versions’ dataset. The numbers represent how many RGB images there are in the set.

	Features				Size (Number of Images)			
	**Top View Only**	**Multiple Colors**	**Multiple T-Shirts**	**Including Desk Class**	**Train**	**Validation**	**Test**	**All**
v1	∘				180	10	10	200
v2	∘	∘			419	23	23	465
v3	∘	∘	∘		614	34	34	682
v4		∘	∘	∘	774	43	43	860

**Table 2:** Examples of the 6 garment states from created garment mesh dataset.

	Flat	Strip	Stack	Fold-2	Fold-4	Fold-s
top	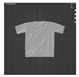	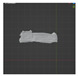	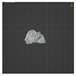	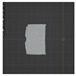	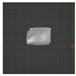	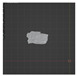
trimetric	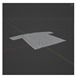	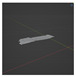	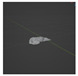	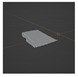	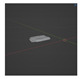	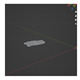

**Table 3:** Average Precision of masks on different test sets of the four models. Mask-RCNN demonstrates better results on multiple t-shirts, while SOLOv2 and light are better on single t-shirt.

Segmentation	AP [IoU 0.50:0.95] (maxDets = 100)								
	**val**	**test**	**v1**	**v2c**	**v3m**	**v3m-a**	**v3m-cd**	**v3m-cs**	**v4sd**
Mask-RCNN [23]	0.814	0.883	0.745	0.846	0.892	0.859	0.836	0.410	0.846
YOLACT [26]	0.833	0.822	0.769	0.875	0.694	0.751	0.688	0.052	0.874
SOLOv2 [27]	0.867	0.882	0.800	0.917	0.797	0.741	0.780	0.084	0.918
SOLOv2-light [27]	0.840	0.882	0.780	0.909	0.749	0.698	0.747	0.064	0.921

**Table 4:** Average Recall of masks on different test sets of the four models. The patterns are similar to those of Average Precision, demonstrating that the training and the models are well-balanced in finding more relevant instances and reducing more irrelevant instances.

Segmentation	AR (maxDets = 100, 300, 1000)								
	**val**	**test**	**v1**	**v2c**	**v3m**	**v3m-a**	**v3m-cd**	**v3m-cs**	**v4sd**
Mask-RCNN [23]	0.872	0.908	0.752	0.892	0.899	0.869	0.877	0.511	0.864
YOLACT [26]	0.877	0.875	0.773	0.917	0.759	0.791	0.725	0.1	0.890
SOLOv2 [27]	0.910	0.912	0.800	0.917	0.828	0.764	0.855	0.152	0.940
SOLOv2-light [27]	0.883	0.913	0.780	0.908	0.807	0.789	0.818	0.109	0.934

**Table 5:** Examples of visualized results on test set v1, v2c, v3ma, and v3mcd.

	v1	v2c	v3ma	v3mcd
input	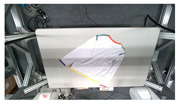	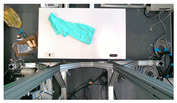	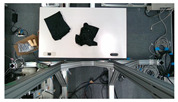	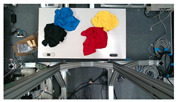
ground truth	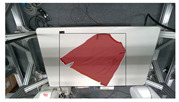	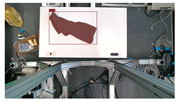	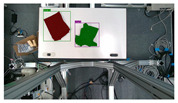	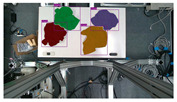
Mask R-CNN	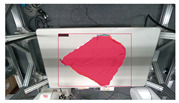	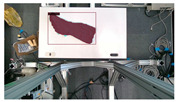	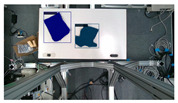	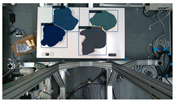
YOLACT	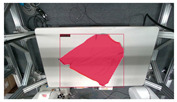	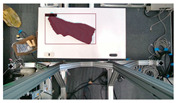	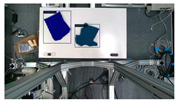	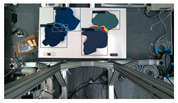
SOLOv2	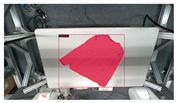	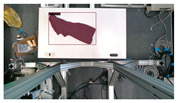	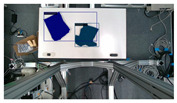	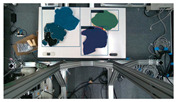
SOLOv2-light	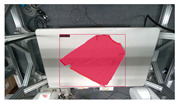	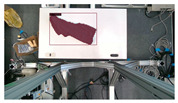	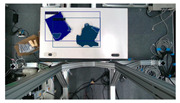	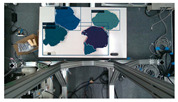

Note: This table continues in Table 6.

**Table 6:** Examples of visualized results on test set v3mcs and v4sd, on a recorded sequence, and directly from a real-time camera input.

	v3mcs	v4sd	Sequence	Camera
input	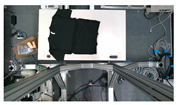	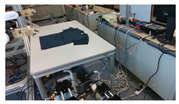	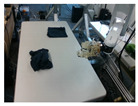	
ground truth	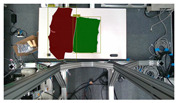	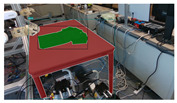		
Mask R-CNN	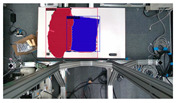	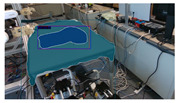	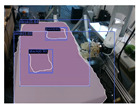	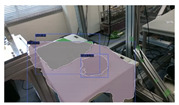
YOLACT	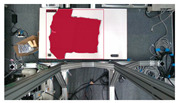	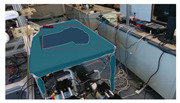	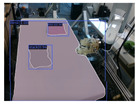	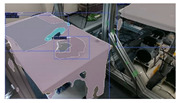
SOLOv2	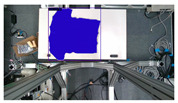	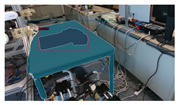	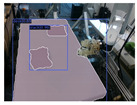	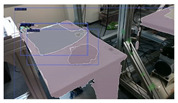
SOLOv2-light	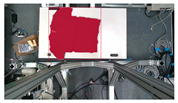	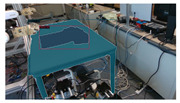	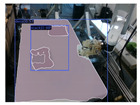	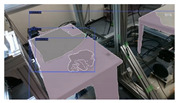

Note: This table continues from Table 5.

**Table 7:** Inference speed of the four models running on self-recorded sequences or with a Realsense D435i camera, evaluated by frames per second (FPS).

	Mask-RCNN	YOLACT	SOLOv2	SOLOv2-Light
sequence	4.62	5.54	4.29	6.00
camera	2.67	4.40	1.63	3.41

**Table 8:** Evaluation of reconstructed objects of each class using the Chamfer distance metric. A smaller Chamfer distance indicates a more accurate reconstruction.

	Flat	Strip	Stack	Fold-2	Fold-4	Fold-s	Desk
Chamfer distance	0.005761	0.000670	0.000051	0.000156	0.000100	0.000084	0.000166

**Table 9:** Example visualizations of the reconstructed mesh from DeepSDF.

	Desk	Flat	Strip	Stack	Fold-2	Fold-4	Fold-s
ground truth	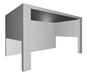	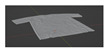	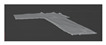	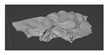	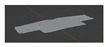	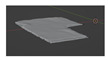	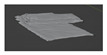
reconstruction	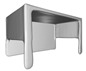	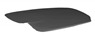	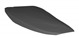	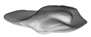	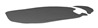	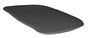	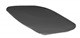

**Table 10:** Visualizations of the integrated SLAM running with a Realsense D435i camera.

	With Only Object Thread	With Only Plane Thread	With Both Integrated
detection	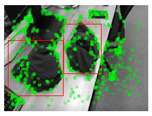	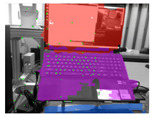	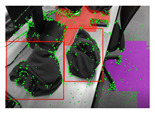
reconstructions	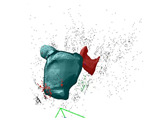	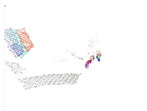	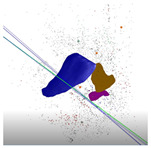

**Table 11:** Speed of each model evaluated by frames per second (FPS) with comparison to their original models.

CAPE [21]	300	YOLACT	4.40		
with only plane thread	1.77	with only object thread	0.69	with both integrated	0.54

## Data Availability

The datasets presented in this article are not readily available because the data are part of an ongoing study. Requests to access the datasets should be directed to zhang.yilin.r8@dc.tohoku.ac.jp.
